# Expression and significance of PIBF combined with Galectin-1 in cervical precancerous lesions

**DOI:** 10.3389/fmed.2025.1660645

**Published:** 2025-10-23

**Authors:** Hong Wang

**Affiliations:** Department of Gynecology, Huai’an First People’s Hospital, The Affiliated Huai’an No. 1 People’s Hospital of Nanjing Medical University, Huai’an, Jiangsu, China

**Keywords:** precancerous lesion, PIBF, Galectin-1, immunohistochemistry, cervical intraepithelial neoplasia

## Abstract

**Objective:**

This study aimed to discuss the significance of PIBF and Galectin-1 in cervical dysplasia tissue.

**Methods:**

The expression of PIBF and Galectin-1 in precancerous tissues of cervical cancer was determined by the immunohistochemical method and ELISA method, and their clinical significance was analyzed.

**Results:**

PIBF and Galectin-1 levels in cervicitis and CIN1 tissues showed no statistically significant difference; the levels of PIBF and Galectin-1 in CIN2 and CIN3 tissues were higher than those in cervicitis and CIN1, and the differences were statistically significant when compared with other groups in the CIN3 group.

**Conclusion:**

Compared with the normal cervical lesion tissue, the levels of PIBF and Galectin-1 in the precervical lesion tissue were significantly increased, suggesting that their elevated levels are significantly associated with the progression of cervical intraepithelial neoplasia.

## 1 Introduction

Cervical cancer, posing a huge threat to women’s health, is one of the most prevalent malignant tumors in gynecology (1). Statistics display that in China, approximately 131,500 new cases of cervical cancer and 30,000 deaths per year (2). Due to the widespread implementation of cervical cancer screening, the mortality of cervical cancer in the United States lessened from 2.8/100,000 in 2000 to 2.3/100,000 in 2015. As a developing country, cervical cancer’s prevalence in China is six times as high as in developed countries, 80% of the patients are diagnosed with invasive cancer. It takes approximately 8–10 years for cervical precancerous lesions to develop into cervical cancer. Therefore, how to better manage patients with cervical precancerous lesions during this time has become a research hotspot (3). It is known that cervical cytology combined with human papillomavirus (HPV) testing plays an important role in screening cervical cancer. Studies have found that cervical cancer caused by HPV16 infection accounts for 55% of all cervical cancer. Cervical cancer caused by HPV18 infection accounts for approximately 20% of all cervical cancer patients, and the remaining high-risk HPV infection accounts for approximately 25%. At present, the prevention of cervical cancer in China is based on HPV testing combined with cytological testing, although the missed diagnosis rate of combined screening is 7–8% (4). In addition, due to the limitation of their own conditions, some patients cannot undergo cervical disease screening as scheduled, so there are still some patients who choose to see a doctor after the appearance of clinical symptoms. The proportion of screened patients with precancerous lesions accounted for approximately 20% of the total number of screened patients (4). Patients with precancerous lesions need long-term follow-up management after necessary treatment, so the choice of indicators that can better predict and measure the outcome of the disease has become a research hotspot. The progesterone-induced block factor (PIBF) plays an immunoprotective role in maternal-fetal immunity, which can prevent the embryo from being recognized by the maternal immune system, and finally allows the embryo to exist in the mother until delivery (5). Galectin-1, a member of the Galectin-1 family, is widely expressed in cells and tissues and takes part in many kinds of pathological and physiological processes, such as cell apoptosis, adhesion, proliferation, and inflammation. Galectin-1 can predict poor disease status and treatment outcome in hematological malignancies (6). Therefore, we predicted that Galectin-1 could induce immune tolerance and promote disease progression. Recently, Galectin-1 is highly expressed at the maternal-fetal interface and takes a vital part in trophoblast cell differentiation, proliferation, invasion, endometrial receptivity, maternal-fetal immune tolerance, and placental angiogenesis (7, 8).

Progesterone regulates PIBF and Galectin-1, both of which play an immunomodulatory role in the maternal-fetal interface. Galectin-1 deficient mice have a significantly increased rate of embryo loss (9, 10). The PIBF index is significantly lower in patients with preeclampsia and those with pregnancy failure (11, 12). In addition, PIBF and Galectin-1 levels were also decreased in pregnant patients treated with mifepristone (13). Other studies have confirmed that elevated PIBF levels inhibit immune responses and promote tumor progression in tissue specimens of breast cancer, bowel cancer, and liver cancer (14, 15). In ovarian cancer patients, PIBF levels are also elevated, promoting tumor invasion (16, 17). However, the progression from cervical intraepithelial neoplasia to invasive cancer is a multi-step and multifactorial process that is not fully understood. Its progression is jointly influenced by various factors such as persistent HPV infection, host immune response, genetic and epigenetic alterations, and changes in the tumor microenvironment (18). Given the crucial roles of PIBF and Galectin-1 in mediating immune tolerance and promoting tissue remodeling at the maternal-fetal interface, we speculate that they may play similar roles in the immune microenvironment of cervical precancerous lesions, potentially promoting disease progression.

Based on the above studies, we speculate that PIBF and Galectin-1 levels are increased in cervical precancerous lesions, and if their levels are significantly increased, they will play a key role in the management and disease progression of patients with cervical precancerous lesions. The trial was done after gaining approval from the Ethics Committee of Huai’an First People’s Hospital.

## 2 Materials and methods

### 2.1 Basic information

This study used a cross-sectional research design, consecutively enrolling patients who underwent colposcopy at our hospital’s Cervical Disease Diagnosis and Treatment Center between October 2022 and December 2023 and met the inclusion criteria, aged 23–69 (37.2 ± 3.1) years old. The sample size was determined based on the principle of convenience sampling, with a target of collecting 50 specimens per group to ensure sufficient samples for preliminary inter-group comparisons. We acknowledge that the absence of *a priori* statistical power calculation is a limitation of this study, which will be elaborated in the discussion section.

The inclusion criteria for the cervicitis group were as follows: patients who underwent colposcopy biopsy due to clinical suspicion of cervical lesions, but whose pathological examination results only indicated chronic cervicitis, without evidence of cervical intraepithelial neoplasia (CIN) or other specific pathogen infections (such as candida and trichomonas). The remaining three groups (CIN1, CIN2, and CIN3) were grouped based on standard pathological diagnostic criteria. Based on the pathological examination results, the patients were divided into four groups: cervicitis group, CIN1 group, CIN2 group, and CIN3 group, with 50 cases in each group.

### 2.2 Methods

#### 2.2.1 Main reagents

Mouse anti-human PIBF and Galectin-1 monoclonal antibodies and enzyme-linked immunosorbent assay (ELISA) kits were purchased from Beijing Baede Biotechnology Co., Ltd.

The mouse anti-human PIBF monoclonal antibody (clone number: 5H7), mouse anti-human Galectin-1 monoclonal antibody (clone number: EPR28790), and the corresponding human PIBF ELISA detection kit (catalog number: EK-310-43) and human Galectin-1 ELISA detection kit (catalog number: EK-115-96) used in this study were all purchased from Beijing Baede Biotechnology Co., Ltd. In the immunohistochemical detection, the working dilution ratios of primary antibodies for PIBF and Galectin-1 were 1:200 and 1:150, respectively.

#### 2.2.2 Test methods

Immunohistochemistry: After dewaxing and hydration, the paraffin-embedded cervical tissue sections underwent antigen retrieval. Subsequently, 3% hydrogen peroxide solution was used to block endogenous peroxidase activity, followed by incubation with normal goat serum at room temperature to block non-specific binding sites. The sections were then incubated overnight at 4 °C with mouse anti-human PIBF monoclonal antibody and mouse anti-human Galectin-1 monoclonal antibody. The next day, the sections were incubated using a polymer-assisted secondary antibody system for 30 min. Finally, diaminobenzidine was used for color development, hematoxylin was used to counterstain the nuclei, and neutral gum was used for mounting. Antibody validation: The commercial primary antibodies used in this study were validated by the supplier through western blot and/or immunohistochemical methods to ensure their specificity and reactivity. In addition, positive controls (tissues known to express PIBF and Galectin-1, such as human placental tissue) and negative controls (using PBS instead of the primary antibody) were set up in each experiment to confirm the effectiveness of the experimental system. Result interpretation and positive definition: All sections were independently observed and evaluated under an optical microscope by two senior pathologists who were unaware of the clinical pathological data. Disagreements were resolved through consultation. A widely used semi-quantitative scoring method was used to comprehensively assess staining intensity and percentage of positive cells: staining intensity: 0 (no color), 1 (pale yellow), 2 (brownish yellow), and 3 (brownish black). The percentage of positive cells was as follows: 0 (<5%), 1 (5–25%), 2 (26–50%), 3 (51–75%), and 4 (>75%). The final score was obtained by multiplying the two scores: 0 (negative), 1–4 (weakly positive), 5–8 (moderately positive), and 9–12 (strongly positive). In this study, a final score of ≥2 was defined as positive expression for subsequent statistical analysis.

ELISA: An appropriate amount of cervical biopsy tissue was taken and homogenized in pre-cooled PBS, and the supernatant was collected after centrifugation. Instructions of the Human PIBF ELISA Detection Kit and Human Galectin-1 ELISA Detection Kit for operation were strictly followed. The brief steps are as follows: Standard and test samples were added to the wells of an ELISA plate coated with specific antibodies. After incubation and washing, biotinylated detection antibodies were added, followed by horseradish peroxidase-labeled streptavidin. Finally, substrate TMB was added for color development, and the absorbance value of each well was measured at a wavelength of 450 nm using a microplate reader. The concentrations of PIBF and Galectin-1 in the sample were calculated based on the standard curve.

### 2.3 Statistical treatment

Statistical analysis was performed using SPSS 19.0 software. Measurement data conforming to a normal distribution were expressed as mean ± standard deviation (x̄ ± s). One-way analysis of variance (ANOVA) was used for multiple group comparisons. If the ANOVA results indicated statistical significance, the least significant difference (LSD) method was further used for pairwise comparisons. We are aware of the risk of type I error inflation when conducting multiple comparisons, and therefore, Bonferroni-corrected *p*-values are also provided for reference. Enumeration data were expressed as case numbers (percentage), and *χ*^2^ test or Fisher’s exact test (when the theoretical frequency is less than 5) was used for inter-group comparisons. Additionally, pairwise comparison results for measurement data were accompanied by 95% confidence intervals. A *p*-value <0.05 was considered statistically significant.

## 3 Results

### 3.1 Expression of PIBF and Galectin-1

The two proteins were expressed in cervical tissue, and the positive rate of CIN3 was high. Compared with other groups, the difference was significant. The positive rates of each group are given in Tables 1, 2.

**Table 1:** Positive expression rate of PIBF in cervical tissues of each group.

Group	*n*	PIBF positive rate (%)
Cervicitis	50	13.2
CIN1	50	14.3
CIN2	50	34.5^a^
CIN3	50	78.3^b^

Values with different superscript letters are significantly different (*p* < 0.05). No significant difference was found between the cervicitis and CIN1 groups.

a *p* < 0.05 vs. cervicitis and CIN1 groups.

b *p* < 0.05 vs. cervicitis, CIN1, and CIN2 groups.

**Table 2:** Positive expression rate of Galectin-1 in cervical tissues of each group.

Group	*n*	Galectin-1 positive rate (%)
Cervicitis	50	11.3
CIN1	50	13.1
CIN2	50	35.5^a^
CIN3	50	67.2^b^

Values with different superscript letters are significantly different (*p* < 0.05). No significant difference was found between the cervicitis and CIN1 groups.

a *p* < 0.05 vs. cervicitis and CIN1 groups.

b *p* < 0.05 vs. cervicitis, CIN1, and CIN2 groups.

### 3.2 Changes of PIBF and Galectin-1 levels in biopsy tissues

Both molecules were detected in each specimen, and their levels increased concomitantly with the severity of the cervical lesions. No statistically significant difference was observed between cervicitis and CIN1, but significant differences exist between cervicitis and CIN1 and high-grade cervical lesions. The Galectin-1 level was higher than PIBF within the same group, and the level of Galectin-1 increased gradually with the severity of lesions. Notably, no significant difference was observed between the cervicitis group and CIN1, but there were obvious differences between the cervicitis group, the CIN1 group, and the other two groups, which is exhibited in Table 3.

**Table 3:** Changes of PIBF and Galectin-1 levels in biopsy tissues.

Groups	PIBF (nmol/L)	Galectin-1 (nmol/L)
Cervicitis	0.131 ± 0.34	0.211 ± 0.16
CIN1	0.148 ± 0.12*	0.313 ± 0.57a
CIN2	0.376 ± 0.51**	0.678 ± 0.19b
CIN3	0.879 ± 0.17***	0.896 ± 0.43c

(1) * and a refers to CIN1 compared with the cervicitis group; the difference was not statistically significant. (2) ** and b refers to difference between CIN2 and CIN1 was significant. (3) *** and c refers to difference between CIN3 and cervicitis, CIN1, CIN2 was significant.

### 3.3 Expression of PIBF in microscopic tissues

**Figure 1:**
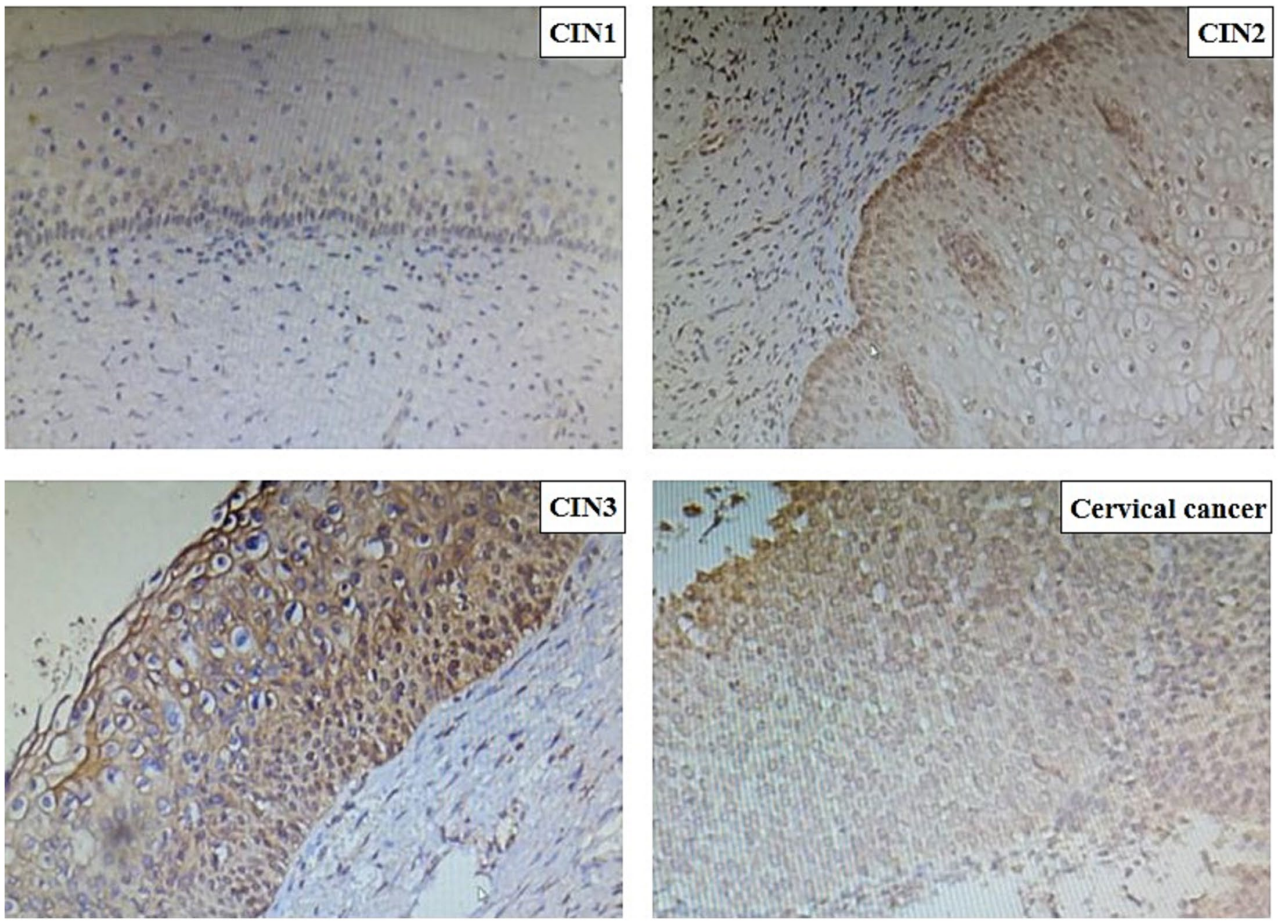
PIBF’s level of cervical tissue in each group in the method of immunohistochemistry.

Immunohistochemical analysis revealed that PIBF expression was significantly upregulated in cervical intraepithelial neoplasia (CIN) tissues compared to the cervicitis group. The staining intensity, along with the nuclear-to-cytoplasmic ratio, increased progressively with the severity of CIN (Figure 1). Notably, in CIN3 lesions, markedly enlarged nuclei and a loss of normal epithelial architecture were observed. Furthermore, disordered cell arrangement was more pronounced in higher-grade lesions.

## 4 Discussion

Cervical cancer is one of the three major malignant tumors in gynecology, with a global incidence of 0.15% according to the WHO (19, 20). Therefore, cervical cancer screening programs are updated year by year, to screen cervical precancerous lesions for early intervention. The missed diagnosis rate of cytology combined with the HPV test is 6–7% (4). How to better manage and monitor the cervical precancerous lesions after medical intervention has become a research hotspot. This study found that the expression of PIBF and Galectin-1 is significantly upregulated in CIN2 and CIN3 lesions, correlating with the severity of cervical lesions. The occurrence of cervical cancer is a complex process involving interactions between viruses, host immunity, and the microenvironment. Our results suggest that PIBF and Galectin-1 may be important participants in this process. Specifically, they may induce an immune-tolerant microenvironment by suppressing local antiviral immune responses (such as NK cell function), thereby facilitating the persistent infection of high-risk HPV and the clonal proliferation of diseased cells. This is consistent with the known mechanism of cervical carcinogenesis, where immune evasion is a key step.

PIBF is a 35KDA protein. Studies have shown that PIBF can affect trophoblast invasion during pregnancy, inhibit natural killer cell activity, and regulate the immune response at the maternal-fetal interface, which is characterized by a bias in the immune response toward a TH2-type cytokine profile. It can invalidate the activation of endogenous PIBF, reduce the production of IL-10, increase the production of IFN-*γ*, and exert an immune rejection effect on embryos, leading to abortion (5, 21, 22). PIBF can regulate endometrial receptivity and promote better embryo implantation in the implantation window. Progestin-treated mice can promote PIBF production and secretion of TH2-type cytokines such as IL-10 and IL-4, which have a protective effect on embryos.

As an immunomodulator, Galectin-1 is highly expressed in the embryonic implantation window, and Galectin-1 mRNA and expression continue to increase during 2.5–11.5 days of gestation in mice (23). Galectin-1 can induce the production of dendritic cells and then promote the secretion of IL-10 by regulatory T cells. Studies on PIBF in tumor invasion patterns have shown that PIBF can activate some molecular genes, such as EGF (epidermal growth factor), initiate invasion signals, secrete proteins such as MMP-9 and MMP-2, and then bind to the corresponding receptors to activate the whole signal transduction pathway (24). If anti-PIBF therapy is given at approximately 10 days of gestation, it results in a higher rate of abortion. In experimental rats, PIBF protects the pregnancy process by inhibiting NK cell activity, mainly NK cell degranulation (25, 26). Galectin-1 inhibits the secretion process of cytotoxic cells and promotes renal cancer cells to migrate through affecting Fas–Fas ligand (27). In addition, the two proteins play a part in cell adhesion, proliferation, migration, and apoptosis. In this research, we found that the expression of PIBF and Galectin-1 in cervicitis and CIN1 patients was significantly lower than that in the high-grade cervical lesions group, indicating that PIBF and Galectin-1 may promote the invasion of lesion cells in cervical lesions, and PIBF and Galectin-1 were significantly increased in CIN3 patients, and the difference was statistically significant. We speculate that PIBF and Galectin-1 exert a synergistic effect at the immunological level to promote local immune tolerance, thereby facilitating disease progression. Therefore, it is reasonable to propose that the combined detection of PIBF and Galetin-1 in the peripheral blood of patients with cervical precancerous lesions can predict the progression of the disease. When the value is increased, it indicates that the disease progresses rapidly, and enough attention should be paid to intervene as soon as possible.

This study has several limitations. First, it is a cross-sectional study, which, although it reveals a significant correlation between the expression levels of PIBF and Galectin-1 and the severity of CIN, cannot establish the direction of causality. Moreover, although the sample size is sufficient for preliminary analysis, no *a priori* statistical power calculation was conducted. Future large-sample prospective cohort studies or mechanistic basic experiments are needed to further verify the predictive value of these biomarkers and elucidate their potential causal effects.

## Data Availability

The datasets presented in this study can be found in online repositories. The names of the repository/repositories and accession number(s) can be found in the article/supplementary material.
